# Electrochemical (Bio) Sensors for Environmental and Food Analyses

**DOI:** 10.3390/bios8030057

**Published:** 2018-06-22

**Authors:** Kevin C. Honeychurch, Martina Piano

**Affiliations:** 1 Centre for Research in Analytical, Materials and Sensors Science, Department of Applied Sciences, University of the West of England, Bristol, Frenchay Campus, Coldharbour Lane, Bristol BS16 1QY, UK; 2 Institute of Bio-Sensing Technology (IBST), University of the West of England, Bristol BS16 1QY, UK

**Keywords:** screen-printed electrodes, pesticides, heavy metals, electrode area, 3-hydroxybutyrate, uric acid, aptamers, personalised medicine, voltammetry, chronocoulometry

## Abstract

In recent years, great progress has been made in the development of sensors and biosensors to meet the demands of environmental and food analysis. In this Special Issue, the state of art and the future trends in the field of environmental and food analyses have been explored. A total of seven papers (three research and four review papers) are included. These are focused on the fabrication and detection of contaminates such as heavy metals, pesticides and food components, including uric acid and 3-hydroxybutyrate. Included in this Issue is a paper dedicated to the experimental determination of the electroactive area of screen-printed electrodes, an important parameter in the development of such sensors.

## 1. Introduction

The aim of this Special Issue of Biosensors, “Electrochemical (Bio) Sensors for Environmental and Food Analyses” is to report recent developments and advances in sensors and biosensors to meet the demands of environmental and food analysis. Its objective is to collect a series of articles which show the developments and applications of both electrochemical sensors and biosensors in this area. The complexity of the environment offers a number of analytical challenges; challenges that need to be met if we are to be able to provide clean drinking water and food, as well as to safeguard environmental quality for ourselves and future generations. Presently, many monitoring regimes are focused on the collection of samples and their subsequent analyses at a centralised laboratory; systems, which, by their nature, have an inherent lag-time, utilise expensive instrumentation, and require highly-trained staff for their implementation. The application of electrochemical sensors and biosensors has shown the possibility of economic, rapid, and decentralised testing of complex samples, carried out by relatively untrained individuals at the point-of-need. Analyses of food and the environment offer large potential markets and opportunities for these devices; however, there are a number of both technical and commercial issues that need to be addressed before these devices can have a significant role.

This Special Issue is comprised of three research and four review articles focused on electrochemical sensors and biosensors for food and environmental analysis. Here Stozhko et al. [1] developed a sensor for the determination of uric acid in serum and milk. They have described the mechanism of uric acid as electro-oxidation occurring on the surface of gold nanoparticles uncomplicated by catalytic steps. The application of gold nanoparticles was found to lower the overvoltage and also increased current of uric acid oxidation. These effects were found to be dependent on particle size and not on the particle preparation method employed. Using a screen-printed electrode (SPE) modified with gold nanoparticles (5 nm) and Nafion a significantly improvement in resolution and sensitivity was reported.

Additionally, Martínez-García et al. [2] reported a biosensor for 3-hydroxybutyrate based on the immobilisation of 3-hydroxybutyrate dehydrogenase at a SPE modified with reduced graphene oxide and thionine. After addition of 3-hydroxybutyrate or the sample in the presence of NAD^+^ cofactor, the enzymatically generated NADH could be determined amperometically allowing for the determination of 3-hydroxybutyrate.

Of interest for the development of SPE devices not just in the food environment sector is knowledge of the electroactive area of the electrode. García-Miranda Ferrari et al. [3] have utilized cyclic voltammetry and chronocoulometry to determine the real electrochemical active area of a SPE. Here they have highlighted the various experimental and mathematical parameters that are need to be considered and controlled in order to obtain useful measurements of the electroactive area.

In the first of the review articles in this Special Issue, Kozitsina et al. [4] have reviewed sensors based on bio and biomimetic receptors for medical diagnostic, environment, and food analysis. They have postulated that the field of analytical chemistry is developing in two main areas: firstly in the automation and creation of devices that allow for the simultaneously analysis of a large number of samples without the participation of the operator. Secondly, in the development of portable miniature devices for personalised medicine and the monitoring of the human habitat. Their review focused on transducers, receptors, techniques of immobilising the receptor layer, signal generation and detection, and methods for increasing sensitivity and accuracy. Examples of the application of bio- and chemical sensors are given along with descriptions of new power supplies and miniaturisation paths, wearable and printed sensors.

Hernandez-Vargas et al. [5] have reviewed the development of SPEs based biosensors; describing recent developments made in the determination of new and emerging pollutants, such as; fluoxetine, carbamazepine, diphenhydramine tetracycline, erythromycin and sulfamethoxazole. The determination of heavy metals, pesticides and anions such as phosphate and nitrate are also reviewed.

Aptamer based sensors for food and environmental applications have been reviewed by Mishra et al. [6]. Here they show that these offer a number of advantages allowing for the development of sensors which are both sensitive and selective. The application of these devices for the determination of heavy metals, pesticides and possible future developments are also discussed.

Bucur et al. [7] have reviewed recent developments in the application and fabrication of enzyme inhibition based biosensors for pesticide determination. Recent advances in enhancing the sensitivity and selectivity of these devices by the application of nanomaterials and novel mutant enzymes in array-type sensor formats is given. The use of these with chemometric methods for data analysis are then reviewed. Their review also reports on the progress made in the application of solar cell technology that could allow for the use of photosynthetic enzymes for the determination of a number of herbicides.
